# Sodium and Potassium Intakes and Their Ratio in Adults (18–90 y): Findings from the Irish National Adult Nutrition Survey

**DOI:** 10.3390/nu12040938

**Published:** 2020-03-28

**Authors:** Eoin Morrissey, Miriam Giltinan, Laura Kehoe, Anne P. Nugent, Breige A. McNulty, Albert Flynn, Janette Walton

**Affiliations:** 1 Department of Biological Sciences, Cork Institute of Technology, T12928 Cork, Ireland; 2 School of Food and Nutritional Sciences, University College Cork, T12 K8AF Cork, Ireland; 3 UCD Institute of Food and Health, University College Dublin, Belfield, D04 V1W8 Dublin, Ireland; 4 Institute for Global Food Security, School of Biological Sciences, Queens University Belfast, Belfast BT7 1NN, Northern Ireland, UK

**Keywords:** sodium, salt, potassium, sodium to potassium ratio, hypertension

## Abstract

An individual’s sodium to potassium intake ratio (Na:K) has been shown to be an important predictor of hypertension. The aim of this study was to estimate the mean 24 h urinary Na, K and Na:K of Irish adults and to identify the foods that determine Na:K in a nationally representative sample of Irish adults. This study was based on data from the Irish National Adult Nutrition Survey (2008–2010) (NANS), which collected spot urine samples and dietary data in a nationally representative sample of Irish adults aged 18+ years. The mean urinary molar Na:K of Irish men and women was 1.90 and 2.15, respectively, which exceed target molar ratios of ≤1.0 and ≤2.0. The mean estimated 24-h urinary excretion of Na was 4631 mg for men and 3525 mg for women, which exceed target maximum population intakes for all gender and age groups. The mean estimated 24-h urinary excretion of K was 3894 mg for men and 2686 mg for women, with intakes in women of all ages and older men (65+ years) below current recommendations. The key foods positively associated with a lower Na:K were fruits, vegetables, potatoes, breakfast cereals, milk, yogurt and fresh meat, while the foods negatively associated with a lower Na:K were breads, cured and processed meats and butters and fat spreads. Strategies to reduce sodium and increase potassium intakes are necessary to lower population Na:K, which may help to reduce the burden of hypertension-related diseases in the Irish population.

## 1. Introduction

A diet low in sodium (Na) and high in potassium (K) is widely recommended as a strategy to lower blood pressure and to reduce the risk of cardiovascular disease (CVD), as high blood pressure (hypertension) is a primary risk factor for CVD [1]. CVD is one of the leading causes of morbidity and mortality in the world, accounting for one-third of deaths both in Ireland and globally [2]; hence, efforts to reduce the proportion of the population with hypertension are of great public health importance. 

The European Food Safety Authority (EFSA) has set an adequate intake (AI) for sodium of 2.0 g/d (equivalent to 5 g/d salt) for adults of all ages, based on a sufficient requirement to maintain sodium balance and at levels associated with a reduced risk of CVD [3]. While meeting the AI for sodium is considered beneficial for health, the Food Safety Authority of Ireland (FSAI) and the UK Scientific Advisory Committee on Nutrition (SACN) have set target maximum population salt intakes of <6 g/d [4,5]. With regard to potassium, the EFSA has set an AI of 3.5 g/d for adults of all ages [6], based on studies showing the blood pressure lowering effects of potassium, with intakes below 3.5 g/d being associated with a higher risk of stroke [7].

While the individual effects of sodium and potassium on blood pressure have been long established, there is a consensus that an individual’s sodium to potassium intake ratio (Na:K) is a more important predictor of hypertension than either sodium or potassium intake alone [1,8,9]. This hypothesis is supported by a large body of evidence including findings from INTERSALT; a large world-wide epidemiological study (consisting of over 10,000 adults aged 20–59 years across 32 countries). This study found that reductions in Na:K had a greater effect on reducing blood pressure than the individual effects of sodium or potassium alone and suggested that lowering of population Na:K would lead to reductions in blood pressure that would enable significant reductions in CVD and mortality rates [9]. For individuals, the World Health Organisation (WHO) recommend a sodium intake <2000 mg/d and a potassium intake >3510 mg/d [10,11], which would yield a Na:K of ≤0.6 mg/mg (≤1.0 mmol/mmol measured by urinary excretion). This ratio of intake is considered beneficial for health and has also been related to a reduction in stroke risk [11,12]. While a molar Na:K ≤1.0 is preferable, compliance to this recommendation is low and recent studies have shown that molar intake ratios between 1.0 and 2.0 may lower CVD risk in adults [13,14,15,16,17,18]. Therefore, a molar intake ratio ≤2.0 has been suggested as a suboptimal goal to lower blood pressure [19].

Measuring urinary excretion of sodium and potassium from multiple 24 h urine collections is considered the gold standard method of measuring an individual’s Na:K [20]. However, for large-scale studies collecting 24-h urine can be costly and time consuming [21]. Therefore, many studies collect spot urine samples which can be used to estimate the 24 h Na:K [22] in addition to population sodium and potassium intakes [23]. INTERSALT has estimated Na:K from 24 h urine collections and reported a mean molar Na:K ranging from 0.01 (Yanomamo, Brazil) to 7.58 (Tianjin, China), with the mean molar Na:K of Western populations estimated to be 2.98 [22]. Individual cross-sectional studies of adults have reported a mean molar Na:K ranging from 2.2 to 3.8 across studies, with Na:K greater than 2.0 in all study groups [13,14,15,16,17,18]. The INTERSALT study has also shown that reductions in the mean population urinary Na:K from 3.09 to 1.0 would result in a reduction of population blood pressure of such size (3–5 mmHg) to reduce stroke mortality by 8%–14%, CHD mortality by 5%–9% and all-cause mortality by 4%–7% [9]. The Irish National Adult Nutrition Survey (2008–2010) (NANS) is the most recent study to collect urinary excretion data for Na and K, in addition to detailed dietary intake data for both nutrients in a nationally representative sample of adults in Ireland. The objective of the following study was to use these data to estimate mean 24 h excretions of sodium, potassium and Na:K in a nationally representative sample of Irish adults. An additional aim was to identify food groups which determine a higher or lower dietary Na:K in Irish adults.

## 2. Materials and Methods

### 2.1. The National Adult Nutrition Survey (NANS) Sample

A detailed description of the methodology used in NANS has been reported elsewhere [24,25]. Briefly, the fieldwork phase of NANS was carried out between October 2008 and April 2010, providing a seasonal balance to the data and biological sample collection. A quota sampling approach was adopted using data from the 2006 Irish Census [26] to achieve a nationally representative sample. Eligible participants were community-dwelling adults aged 18 years and over who were not pregnant or lactating. The study was conducted according to the guidelines laid down in the declaration of Helsinki and all procedures involving human participants were approved by the Clinical Research Ethics Committee of the Cork Teaching Hospitals, University College Cork and the Human Ethics Research Committee of University College Dublin (ethical approval code: Ref.: ECM 3 (p) 04/11/08). Written informed consent was obtained from all eligible and willing participants. The final response rate for the survey was 60%. Analysis of the demographic features in this sample has shown it to be a representative sample of Irish adults with respect to age, gender, social class and geographical location when compared to Census data [26]. While participation in the survey did not require provision of a urine sample as an eligibility criterion, all participants were asked whether they were willing to provide one, and 75% (*n* 1121) provided a sample.

### 2.2. Urine Sample Collection, Processing and Analysis

Participants were provided with a sterile container to collect a first void urine sample (minimum, 20 mL; aim, 50 mL) and instructed to fast for 12 h prior to obtaining the sample. An icepack was also provided to keep the sample chilled until same day collection by the researcher. Samples were transported to laboratories at University College Cork or University College Dublin, where they were processed and stored at −20 °C until analysis. Urine samples were typically obtained either at the same time as the 4 days of dietary assessment or within a 2-week period. Urinary Na and K were measured by the Randox Rx Daytona with an ion-selective electrode. The quality control material used was Randox QC urine with assigned values for Na and K, which were measured at both low and high levels. The quality assurance used was the Randox International Quality Assessment Scheme. The interassay coefficient for Na was ≤5.0% and for K was ≤4.1%.

### 2.3. Food Consumption Data

A 4-day semi-weighed food diary was used to collect food and beverage intake data. Participants (*n* = 1500) were visited by a trained fieldworker three times during the recording period inclusive of one detailed training session prior to study commencement. Participants were asked to record detailed information (at the brand level) on the types and amounts of all foods, beverages and nutritional supplements consumed over the 4-day period. Details of recipes, leftovers, and (where applicable) cooking method used were also recorded. Participants were also encouraged to keep food labels to provide further information on the nutrient composition of the consumed foods. A hierarchal method [27] was used to quantify the amount of each food/beverage consumed. This included direct weighing of the food by participants, provided by manufacturer’s information, use of a photographic food atlas [28], standard food portion sizes [29] and household measures.

Intakes of energy, sodium and potassium were estimated using WISP^©^ nutritional analysis software which includes data from McCance and Widdowson’s ‘The Composition of Foods’ sixth edition [30] and supplemental volumes [31,32,33,34,35,36,37,38,39]. Modifications to this nutrient database were carried out at the time of the survey and included addition of all nutritional supplements, fortified foods, recipes and generic Irish foods that were recorded during the survey period [40]. Sodium values were updated using analytical data provided by the Food Safety Authority of Ireland and current manufacturer’s information at the time of the survey (derived from product labels). For dietary analyses, salt added at the table or in recipes was not accounted for. For foods that may be cooked in salted or unsalted water such as vegetables and pasta, the default of ‘boiled in unsalted water’ was used.

### 2.4. Data Analyses

#### 2.4.1. Urinary

Mean 24 h excretions of sodium and potassium intake were estimated by correcting the mean Na and K concentrations in the spot urine samples for gender-specific 24-h urine volume estimations for Irish adults derived from a study by Perry et al. [41] which has been shown to provide adequate population-level estimates of sodium and potassium intakes [23]. The 24-h Na excretions were compared to the EFSA AI of 2.0 g/d [3] and the 24-h K excretions were compared to the EFSA AI of 3500 mg/d [7]. Urinary molar Na:K was calculated for each participant as Na (mmol/L)/K(mmol/L), with a value of 0.6 being subtracted from each individual value to correct for timing bias associated with circadian rhythms [42]. Compliance with Na:K target molar ratios was examined by assessing the proportion of the population complying with the WHO guideline of ≤1.0 and the suggested suboptimal target of ≤2.0 [11,19]. 

#### 2.4.2. Dietary

Usual intake distributions of sodium and potassium and dietary molar Na:K were estimated using the validated National Cancer Institute (NCI) method [43] using SAS Enterprise Guide^©^ Version 6.1 (SAS Institute Inc., Cary, NC, USA). The NCI method has been implemented in SAS macros (version 2.1) which were downloaded from the website www.riskfactor.gov/diet/usualintakes/macro.html (date of download: July 2015). SPSS Version 21 for Windows^TM^ (SPSS, Inc.) was used for all further dietary analyses. The key sources of sodium and potassium were calculated by the mean proportion method as defined by Krebs-Smith et al. [44]. This method provides information about the sources that are contributing to the nutrient intake “per person” and is the preferred method when determining important food sources of a nutrient for individuals in the population group as opposed to investigating the sources of a nutrient within the food supply. The contribution of discretionary salt to total salt intake in the Irish population was crudely estimated for each gender and age group (18–35, 36–50, 51–64 and 65+ years) by examining the difference between mean dietary sodium intake and mean urinary sodium excretion. Dietary molar Na:K was calculated for each individual as the mean daily intake of sodium (excluding discretionary salt) divided by the mean daily intake of potassium. In order to examine the foods/food groups that are associated with a lower dietary Na:K in Irish adults, the population was divided into thirds (low, medium, high) according to their dietary Na:K (stratified by gender and age group). Differences in intakes of sodium, potassium, dietary Na:K and food group intakes between participants with low and high Na:K were assessed using a Mann–Whitney *U* test and Kruskal–Wallis test (for non-normally distributed data). To minimise type 1 errors (as a result of multiple testing), the Bonferroni adjustment was used, and therefore intakes were considered to be statistically different if *P* < 0.001.

## 3. Results

### 3.1. Urinary Data

Table 1 describes the mean 24 h urinary excretions of Na, salt equivalents, K, urinary molar Na:K and prevalence of urinary Na:K ≤1.0 and ≤2.0 in Irish adults based on first void spot urine samples corrected for gender-specific population 24 h urine volume estimations. The mean 24-h urinary excretion of Na was 4631 mg for men and 3525 mg for women, equivalent to a salt excretion of 11.6 g for men and 8.8 g for women, which are higher than the EFSA AI of 2.0 g/d for sodium (salt equivalent: 5 g/d) and target maximum population salt intakes (<6 g/d) [3,4,5]. The mean 24-h urinary excretion of K was 3894 mg for men and 2686 mg for women compared to the EFSA adequate intake of 3500 mg [7]. The mean urinary molar Na:K was 1.90 for men and 2.15 for women. When assessing compliance with target molar ratios, 31% of men and 25% of women had a urinary Na:K ≤1.0, while 60% of men and 57% of women had a molar Na:K ≤2.0.

### 3.2. Dietary Data

#### 3.2.1. Dietary Intakes of Sodium and Potassium

Mean dietary intakes of sodium (excluding discretionary salt) and potassium are reported as absolute intakes (mg/d) and corrected for energy intake (mg/10 MJ) for total population 18–90 y and by gender and age group (Table 2). The mean dietary sodium intake was 2877 mg/d in men and 2134 mg/d in women. There was no difference in sodium intake between men and women in the total population after adjusting for energy (men 3022 mg/10 MJ, women 3077 mg/10 MJ). The mean dietary potassium intake was 3417 mg/d in men and 2703 mg/d in women. When adjusted for energy intake, women of all ages had higher potassium intakes than men (men 3621 mg/10 MJ, women 3897 mg/10 MJ).

#### 3.2.2. Dietary Sources of Sodium and Potassium Intake

**Figure 1:**
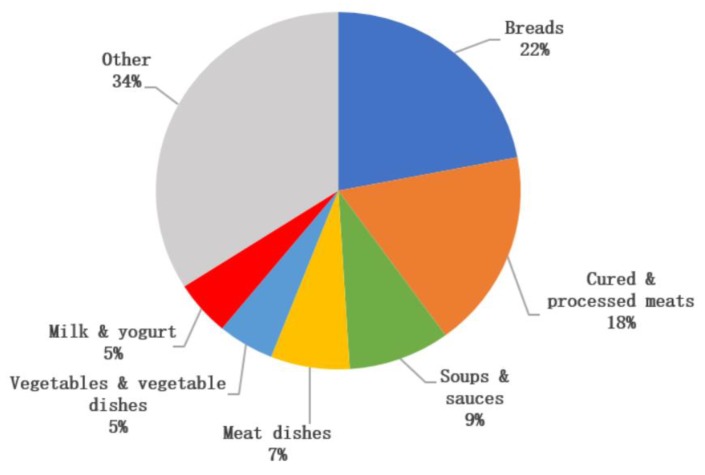
Key sources of sodium intake (excluding discretionary salt) in Irish adults.

**Figure 2:**
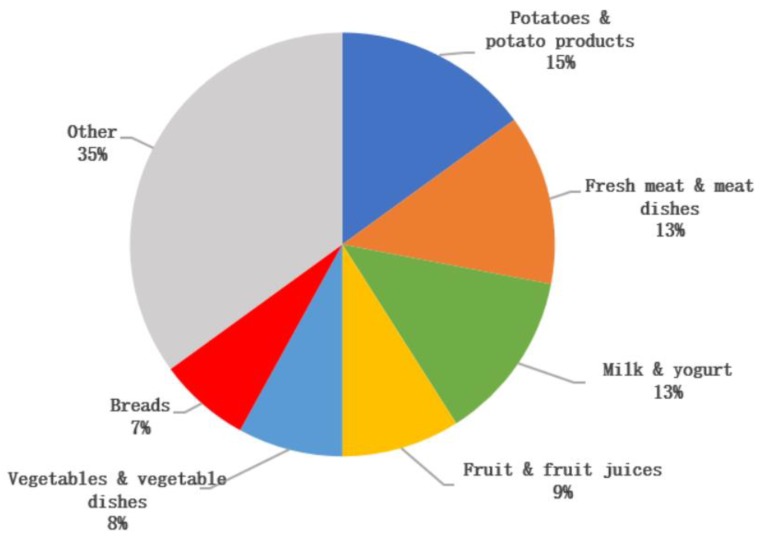
Key sources of potassium intake in Irish adults.

The key sources of sodium were ‘breads’ (22%), ‘cured and processed meat’ (18%), ‘soups and sauces’ (9%), ‘meat dishes’ (7%), ‘vegetables and vegetable dishes’ (5%) and ‘milk and yogurt’ (5%) (Figure 1). The key sources of potassium were ‘potatoes and potato products’ (15%), ‘fresh meat’ and ‘meat dishes’ (13%), ‘milk and yogurt’ (13%), ‘fruit and fruit juices’ (9%) ‘vegetables and vegetable dishes’ (8%), and ‘breads’ (7%) (Figure 2). Discretionary salt (i.e., salt added at the table or in cooking) was estimated to account for 30%–45% (range across gender and age groups) of total salt intake.

#### 3.2.3. Foods Associated with A Lower Na:K

Table 3 reports food group intakes across tertiles of dietary Na:K. Food groups that were negatively associated with a low Na:K were ‘breads’, ‘cured and processed meat’ and ‘butters, spreading fats and oils’. Food groups that were positively associated with a low Na:K were ‘breakfast cereals’, ‘milk and yogurt’, ‘potato and potato products’, ‘vegetables and vegetable dishes’, ‘fruit and fruit juices’, ‘fresh meat’ and ’nuts and seeds, herbs and spices’.

## 4. Discussion

The aim of this study was to estimate mean 24-h excretions of sodium and potassium (including Na:K) in a nationally representative sample of Irish adults. The mean 24-h urinary Na excretion was 4631 mg/d for men and 3525 mg/d for women, with mean sodium intakes higher than the EFSA AI of 2.0 g/d (5 g/d salt) and target maximum population intakes (<6 g/d) for all age groups of men and women [3,4,5]. The mean urinary 24-h K excretion was 3894 mg/d for men and 2686 mg/d for women; mean intakes in women of all age groups and men aged >65 years were below the AI of 3500 mg/d, while all age groups of younger men (<65 years) had mean intakes above the AI [7]. The key finding from this study was that these high Na and low K excretions resulted in a mean urinary molar Na:K of 1.90 for men and 2.15 for women, with over 72% of Irish adults exceeding the target urinary molar Na:K of ≤1.0 and almost half (41%) exceeding a molar Na:K of ≤2.0.

The 24-h urinary sodium and potassium values have been measured in adults in nationally representative surveys in Ireland and the UK (Ireland: Survey of Lifestyle, Attitudes and Nutrition (SLÁN) (2007) and UK: National Diet and Nutrition Survey (NDNS) (2008–2012)) and also in cross sectional studies in Italy, Greece, South Africa, New Zealand, Japan and New York City. The estimated mean daily sodium intakes of adults in Ireland and the UK ranged from 3404 to 4290 mg/d in men and 2668 to 3040 mg/d in women [41,45,46,47], while the estimated potassium intakes ranged from 3128 to 3788 mg/d in men and 2562 to 3034 mg/d in women [41,48]. The mean urinary molar Na:K of the adult populations from the cross sectional studies reviewed ranged from 2.2 to 3.8 across studies—greater than the target molar ratios of ≤1.0 and ≤2.0 across all population groups [13,14,15,16,17,18]. Despite variations in methodologies and study samples, these studies suggest that Irish adults have higher sodium and potassium intakes compared to adults in the UK, in addition to a higher mean urinary Na:K than adults in New Zealand, but a lower mean urinary Na:K than adults in Italy, Greece, South Africa, Japan and New York City [13,14,15,16,17,18].

Successful implementation of strategies to reduce population Na:K may reduce the burden of CVD on public health. The Trials of Hypertension Prevention (TOHP) study estimated sodium and potassium intakes in adults (30–54 years) from multiple 24-h urine collections and analysed CVD occurrence in 10–15 years of post-trial follow up [19]. The findings of this study showed a direct association between Na:K and CVD risk [19], highlighting the potential benefits of any reductions in population Na:K. Similarly, findings from INTERSALT study suggest that a reduction in the mean population urinary Na:K from 3.09 to 1.0 would enable a mean reduction in population systolic blood pressure of 3.36 mmHg [9]. It is thought that reductions in population blood pressure of such size (3–5 mmHg) would reduce stroke morality by 8%–14%, CHD mortality by 5%–9% and all-cause mortality by 4%–7% [9]. Despite some studies reporting a j-shaped relationship between sodium intake and diseases rate [49,50], data from 23 to 26 years of post-trial follow up from the TOHP study found a direct linear relationship between sodium intake and mortality occurrence [51]. The j-shaped relationship found in studies may be explained by measurement bias or reverse causation [52,53], and thus potential adverse health effects due to low sodium intakes at a population level appear unlikely and should not stall efforts to reduce population sodium intakes and Na:K.

The detailed dietary data in the current study allowed us to identify the key dietary sources of sodium and potassium intakes in Irish adults. Key contributors to sodium intakes were primarily processed foods, such as cured and processed meats, breads and soups and sauces, which is similar to the key dietary sources of sodium reported among adults globally [48,53,54,55]. The key contributors to potassium intakes in Irish adults were similar to that reported in other studies, with potatoes, fresh meat, dairy products and fruits and vegetables commonly reported as key dietary sources of potassium in adults [48,54,55]. Of interest, comparison of dietary intakes and urinary excretion values in this study suggests that discretionary salt added at the table or in cooking may account for 30%–45% (range across age groups) of total salt intake in the Irish population. It should be noted that this is a crude estimate for discretionary salt and should be interpreted with caution. However, this should not reduce the importance of implementing strategies to reduce population sodium intakes including that of discretionary salt. Salt reduction programmes such as those in Ireland and the UK have been implemented to achieve gradual, sustained and universal reductions in the salt content of processed and prepared foods such as those identified as key contributors to sodium intakes. These programmes have resulted in significant reductions of up to 60%–70% in the salt content of foods sold in those countries [56,57]. As a result, population salt intakes in these countries have also significantly decreased, with a decrease of over 1.0 g/d recorded in the mean salt intake of Irish adults between 2001 and 2011 [58], while a decrease of 0.9 g/d was observed in the mean salt intake of adults in the UK between 2000 and 2008 [57]. While the success of such programmes is evident, continued engagement from the food industry is necessary if population target salt intakes are to be achieved. Even slight reductions in the salt content of highly consumed categories of foods can potentially lead to a significant reduction in sodium intakes amongst population groups [4].

This study also investigated food groups that are associated with a higher/lower dietary Na:K in Irish adults, which is a critical step that could aid in strategies to change population-level intakes [53]. The foods positively associated with a lower dietary Na:K were ‘breakfast cereals’, ‘milk and yogurt’, ‘potato and potato products’, ‘vegetables and vegetables dishes’, ‘fruit and fruit juices’, ‘fresh meat’ and ‘nuts and seeds, herbs and spices’, while the foods negatively associated with a lower dietary Na:K were ‘breads’, ‘butters, spreading fats and oils’ and ‘cured and processed meat’. Efforts to reduce Na:K in Irish adults and elsewhere should focus on reducing the consumption of food groups found to be negatively associated with a low Na:K and increasing the consumption of the foods found to be positively associated with a low Na:K [59]. It is important to note that food groups associated with a lower Na:K are in line with recommendations from the Food-Based Dietary Guidelines (FBDGs) for those in the WHO European region, such as dairy, cereals, vegetables and fruit, while food groups associated with a higher Na:K are those recommended in moderation or with consumption limits such as butters and fat spreads and processed meats. Individual efforts to reduce salt intake have been shown to be often ineffective [60], emphasizing a need for increased population compliance with the FBDGs which are designed to provide advice on foods, food groups and dietary patterns to provide all of the required nutrients in adequate amounts to promote health and prevent chronic diseases. Further efforts to reduce the salt content of products will aid in the reduction of salt intakes and such reductions, along with a parallel increase in the consumption of potassium-rich foods such as fruits and vegetables, may aid in lowering population Na:K [60].

The main strengths of this study are that it is based on a nationally representative sample of the Irish population and includes urinary data to complement detailed dietary data for sodium and potassium intake estimates. The detailed dietary intake data were collected at the brand level for each food consumed and sodium values were updated with analytical data on Irish foods. While morning spot urine samples may overestimate sodium excretion and underestimate potassium excretion, appropriate bias correction was applied in this study to estimate the mean Na:K of Irish adults.

## 5. Conclusions

This study provided estimates of Na:K intake in a nationally representative sample of Irish adults. A high sodium intake coupled with low potassium intake (in some age-gender groups) resulted in a mean urinary molar Na:K of 1.90 and 2.15 for Irish men and women, respectively. Consistent with other studies, these estimated Na:K exceed target molar ratios of ≤1.0 and ≤2.0. The key foods found to be positively associated with a lower Na:K were fruits, vegetables, potatoes, breakfast cereals, milk, yogurt and fresh meat, while the foods found to be negatively associated with Na:K were breads, cured and processed meats and butters and fat spreads. Strategies to reduce sodium and increase potassium intakes are necessary in order to lower population Na:K, which may help to reduce the burden of CVD in the Irish population and elsewhere.

## Figures and Tables

**Table 1:** The 24 h urinary excretions of sodium, salt and potassium.

	Urinary Sodium (mg)	Urinary Salt Equivalents (g)	Urinary Potassium (mg)	Na:K (mmol/mmol)		Urinary Molar Na:K ≤1.0 **	Urinary Molar Na:K ≤2.0 ***
	Mean	Mean	Mean	Mean	SD	%	%
Men	4631	11.6	3894	1.90	1.49	31	60
18–35 years	4677	11.7	3775	1.99	1.57	32	56
36–50 years	4693	11.7	4306	1.71	1.36	32	67
51–64 years	4504	11.3	3888	1.82	1.39	31	68
65+ years	4550	11.4	3393	2.17	1.59	25	53
Women	3525	8.8	2686	2.15	1.67	25	57
18–35 years	3925	9.8	2887	2.35	1.92	22	52
36–50 years	3528	8.8	2830	2.01	1.58	30	61
51–64 years	3276	8.2	2505	2.08	1.55	25	58
65+ years	2895	7.2	2139	2.05	1.28	21	57

Urinary Na:K (mmol/mmol) and prevalence of urinary Na:K ≤1.0 in Irish adults by age group and gender based on spot urine samples (*n* = 1121).* Corrected for gender-specific 24-h urine volume estimations derived from a study by Perry et al. (men: 1.97 L; women: 1.67 L). ** World Health Organisation (WHO) recommendation (molar Na:K ≤1.0) (WHO guidance on potassium at least 3510 mg per day, on sodium less than 2000 mg per day). *** Shown to exhibit lower CVD risk.

**Table 2:** The mean daily intakes of sodium, salt and potassium and dietary molar Na:K in Irish adults (*n* =1500).

	*n*	Sodium				Salt				Potassium				Dietary Na:K	
		mg/d		mg/10 MJ		g/d		mg/10 MJ		mg/d		mg/10 MJ		(mmol/mmol)	
		mean	SD	mean	SD	mean	SD	mean	SD	mean	SD	mean	SD	Mean	SD
Total population	1500	2501	737	3025	456	6.25	1.85	7.56	1.14	3055	886	3761	685	0.89	0.89
18–35 years	531	2687	758	3053	459	6.71	1.91	7.63	1.15	3036	945	3450	584	0.97	0.22
36–50 years	437	2508	733	3049	452	6.27	1.85	7.63	1.13	3098	873	3839	650	0.88	0.20
51–64 years	306	2363	670	2926	439	5.91	1.68	7.31	1.10	3138	851	3971	678	0.81	0.19
65+ years	226	2236	653	3045	460	5.59	1.64	7.61	1.15	2904	790	4054	699	0.83	0.20
Men	740	2877	696	3022	456	7.19	1.76	7.56	1.14	3417	864	3621	624	0.92	0.21
18–35 years	276	3073	698	2988	450	7.67	1.77	7.47	1.13	3522	872	3432	585	0.96	0.22
36–50 years	205	2932	671	3080	454	7.33	1.70	7.71	1.14	3466	853	3670	608	0.92	0.21
51–64 years	153	2671	640	2925	440	6.67	1.62	7.31	1.10	3402	848	3768	624	0.85	0.19
65+ years	106	2562	619	3137	458	6.40	1.56	7.84	1.15	3070	794	3803	623	0.91	0.20
Women	760	2134	572	3027	455	5.33	1.43	7.57	1.14	2703	756	3897 *	714	0.86	0.22
18–35 years	255	2270	579	3123 *	458	5.67	1.46	7.81	1.15	2510	710	3470 *	583	0.99	0.22
36–50 years	232	2134	561	3022 *	449	5.33	1.41	7.55	1.12	2773	754	3988 *	649	0.84	0.19
51–64 years	153	2056	548	2926	437	5.14	1.37	7.32	1.09	2875	767	4174 *	669	0.77	0.18
65+ years	120	1948	536	2965 *	446	4.87	1.34	7.40	1.11	2758	756	4276 *	688	0.76	0.18

Na intake is estimated from food sources only and does not account for discretionary sources (added at the table or during cooking). * Denotes statistically significant difference (*P* < 0.001) from that of men within the column via a Mann–Whitney *U* test.

**Table 3:** Dietary intake of sodium, potassium, dietary molar Na:K and food group intakes in Irish adults (*n* = 1500) by tertile of dietary molar Na:K (stratified by age group and gender).

	Low		Medium		High		*P* Value
	*n* = 499		*n* = 502		*n* = 499		
	Mean	SD	Mean	SD	Mean	SD	
Dietary Na:K (mmol/mmol)	1.0	0.2	1.4	0.2	1.9	0.3	0.000
Mean daily intake of sodium (mg)	1993	705	2556	782	2947	938	0.000
Mean daily intake of potassium (mg)	3432	1163	3112	887	2621	816	0.000
Food group intakes (g/d)							
Grains, rice, pasta and savouries	60.2	75.8	59.8	68.8	59.6	69.6	0.756
Breads	89.5	54.2	118.5	59.0	138.6	65.4	0.000
Breakfast cereals	81.5	95.2	61.4	79.4	42.8	60.7	0.000
Biscuits, cakes and pastries	28.7	34.6	31.3	35.3	28.7	35.0	0.165
Milk	244	213	223	188	163	143	0.000
Yogurt	37.8	53.7	38.6	59.0	20.5	40.6	0.000
Creams, ice-creams and chilled desserts	22.5	38.1	22.2	36.9	17.9	32.9	0.042
Cheeses	11.0	15.4	14	17.6	15.8	19.8	0.002
Butter, spreading fats and oils	10.7	11.0	14.3	13.8	20.9	21.0	0.000
Eggs and egg dishes	15.3	23.2	15.9	21.9	18.7	25.9	0.069
Potatoes and potato products	138	96.5	125	78.5	98.1	75.1	0.000
Vegetables and vegetable dishes	132	101	112	72.0	98.5	68.8	0.000
Fruit	135	135	95.9	109	28.8	76.6	0.000
Fruit juices	62.3	104	52.0	84.8	34.2	68.3	0.000
Fish and fish dishes	33.0	44.6	29.2	39.5	24.0	37.0	0.002
Meat and meat dishes	166	96.1	181	97.2	189	103	0.001
Cured and processed meat	38.6	39.4	57.3	51.4	80.5	61.6	0.000
Fresh meat	68.9	59.8	57.0	48.4	47.9	45.3	0.000
Meat dishes	58.6	77.9	66.5	79.4	61.1	77.6	0.138
Beverages	1628	936	1507	823	1368	787	0.098
Alcoholic beverages	351	623	322	589	228	423	0.021
Tea	449	429	470	403	419	386	0.056
Coffee	144	236	117	191	111	207	0.016
Other beverages	683	666	598	609	609	621	0.086
Sugars, confectionery, jams and savoury snacks	28.7	29.9	30.2	26.4	29.7	27.8	0.201
Soups and sauces	56.9	75.1	56.5	64.1	56.6	65.9	0.323
Nuts and seeds, herbs and spices	4.1	12.0	2.8	8.8	1.8	5.9	0.000

*P* < 0.001 denotes significance differences across tertile groups via a Kruskal–Wallis test. Note: values in table are based on dietary assessment and do not include measures of discretionary salt.
